# Research progress on the correlation between estrogen and estrogen receptor on postmenopausal sarcopenia

**DOI:** 10.3389/fendo.2024.1494972

**Published:** 2024-11-21

**Authors:** Chengmei Zhang, Xin Feng, Xue Zhang, Yu Chen, Juan Kong, Yan Lou

**Affiliations:** ^1^ Department of Clinical Nutrition, Shengjing Hospital of China Medical Universty, Shenyang, Liaoning, China; ^2^ Department of Nephrology, Liaoning Electric Power Central Hospital, Shenyang, China; ^3^ Sheng Jing Hospital Affiliated, China Medical University, Shenyang, Liaoning, China; ^4^ The Second Affiliated Hospital of Liaoning University of Traditional Chinese Medicine, Shenyang, Liaoning, China; ^5^ School of Intelligent Medicine, China Medical University, Shenyang, Liaoning, China

**Keywords:** estrogen, estrogen receptor, skeletal muscle, postmenopausal sarcopenia, sex steroid hormone

## Abstract

Estrogen is a necessary sex steroid and potent neuroprotective hormone. It plays a multifaceted role beyond the reproductive system, extending its influence to the brain, skeletal muscle, and other organs. Estrogen’s role in cognition, mood, autonomic regulation, and neuroprotection involves interactions with neurotransmitters, neuromodulators in a distributed manner. Notably, the impact of estrogen on mitochondrial metabolism in skeletal muscle is particularly significant due to a unique modulated bioenergetic profiles, synaptic plasticity, and neuronal health. The deficiency of estrogen in menopause has been linked to changes in brain structure, connectivity, energy metabolism. Therewith, these are crucial factors in cognitive function and the risk of Alzheimer’s diseases. Besides, it leads to endocrine and metabolic dysfunction, resulting in osteoporosis, metabolic syndrome, and a tendency toward decreased muscle mass and strength. Estrogen’s influence on mitochondrial function is particularly relevant to aging, as it affects the production of ATP and the overall metabolic health of the brain. Estrogen decline in women skeletal muscle mass is usually related to sarcopenia, a prevalent disease observed in vulnerable elderly individuals. Therefore, estrogen is considered to play a crucial role in skeletal muscle homeostasis and motor ability, although the exact mechanism remains unclear. This paper reviews the literature on the impact of estrogen on postmenopausal skeletal muscle diseases and the underlying molecular mechanisms, especially in terms of mitochondrial metabolism. In summary, estrogen plays an important role in the health of skeletal muscle in postmenopausal women, and its impact on mitochondrial function and homeostasis offers potential targets for the development of new strategies to treat sarcopenia.

## 1 Introduction

Sarcopenia has recently become a primary focus of research and public health strategies owing to its dramatical impact on patients’ quality of life, medical care expenditure, incidence rate, and mortality (1, 2). A previous cross-sectional study demonstrated that muscle mass in postmenopausal women decreases by 0.6% annually (3) and the decline in skeletal muscle mass is usually associated with muscle wasting syndrome (3). With the reduction in muscle mass, physical activity capacity and the number of metabolically active tissues decrease, increasing the risk of metabolic diseases (4). From European Working Group on sarcopenia definition includes low muscle strength, muscle quantity or mass, and muscle functioning problems (5–7). Among them, changes in muscles, and bones throughout the body often lead to adverse events, such as daily activity disorders, falls, and increased complications, directly reducing the quality of life of patients and increasing the risk of death (7–9). Additionally, muscle metabolism has a significant impact on multiple aspects of muscle function, including muscle development, strength, and the overall health of the musculoskeletal system (10).

Estrogen plays an indispensable role in physiological and pathological processes, especially in skeletal muscle (11). A large number of clinical studies have shown human on the correlation between estrogen levels and muscular strength. The influences of estrogen on muscle physiology by modulating muscle mass, strength, and metabolism (5, 12–14). In some pathological conditions, such as postmenopausal sarcopenia (PS), a lack of estrogen in the body can cause muscle atrophy and weakness. This result highlights the importance of towers in maintaining muscle integrity (15–17). Accordingly, the lack of estrogen in postmenopausal women may reduce their sensitivity to synthetic metabolic stimuli and accelerate muscle loss (18). Estrogen has been shown to enhance muscle protein synthesis and reduce muscle breakdown, thereby preserving muscle mass (19). Furthermore, research has shown that estrogen also plays a role in maintaining muscle strength, particularly in females (20). This study provides significant insights into the relationship between estrogen levels and muscle strength in postmenopausal women, which is highly relevant to our manuscript’s focus (21). Besides, a review discusses estrogen deficiency can lead to decrements in muscle strength from both inadequate preservation of skeletal muscle mass and decrements in the quality of the remaining skeletal muscle (22).

The interplay between estrogen and muscle physiology becomes particularly pronounced under physiological conditions and during the aging process. The synergistic effect of testosterone further amplifies muscle strength, highlighting the complex hormonal interplay in muscle maintenance (23). On another hand, during menopause, estrogen levels decline, leading to alterations in brain structure and connectivity, energy metabolism, and an increased risk of neurodegenerative diseases like Alzheimer’s (24). Besides, the molecular mechanisms underlying estrogen deficiency in sarcopenia, especially in postmenopausal women, involve the dysregulation of muscle bioenergetic signaling (25), which can lead to muscle wasting and weakness.

Currently, some suggestions exhibited that hormone replacement therapy (HRT) can mitigate degenerative changes in skeletal muscles (26, 27), aiming to alleviate the symptoms of estrogen deficiency and improve muscle mass and function in postmenopausal women. However, studies on HRT yield inconsistent findings about how estrogen levels are linked to muscle mass or strength (28, 29). However, before making recommendations on the type, dosage, duration, and duration of hormone replacement therapy, a deeper understanding of the direct and indirect mechanisms of female hormones is necessary. After understanding the specific mechanisms, the aim of this review is to help identify targets for the treatment of sarcopenia in postmenopausal women.

## 2 Postmenopausal sarcopenia

As postmenopausal women age, their hormone levels change, making them more prone to changes in tissue structure and organ function (30). Bone loss often occurs three to five years after menopause, and sarcopenia has a high incidence in postmenopausal women (15). Estrogen is a sex steroid hormone secreted by the ovaries primarily, and estrogens comprise a class of hormones that encompasses estrone (E1), estradiol (17β-estradiol; E2), and estriol (E3) (31). As the reproductive system develops and maintains, it regulates the differentiation of the sexual organs (32–34). However, the function of estrogen extends far beyond the reproductive organs. It also plays a vital role in the physiology of other systems and tissues, including the nervous and cardiovascular, central nervous system, and skeletal muscles (35).

Menopause is associated with a natural decline in estrogen levels, which cause an increase in visceral fat and a reduction in bone density, muscle mass, and strength (19). It has been reported in the literature that the reduction in skeletal muscle mass and strength in postmenopausal women is typically greater than that in age-matched males, especially after 55 years of age (18). Specifically, after this age, grip strength and knee extensor strength in men decreased by 23% and 17.4%, respectively, whereas those in women decreased by 40.2% and 28%, respectively (36).

**Figure 1:**
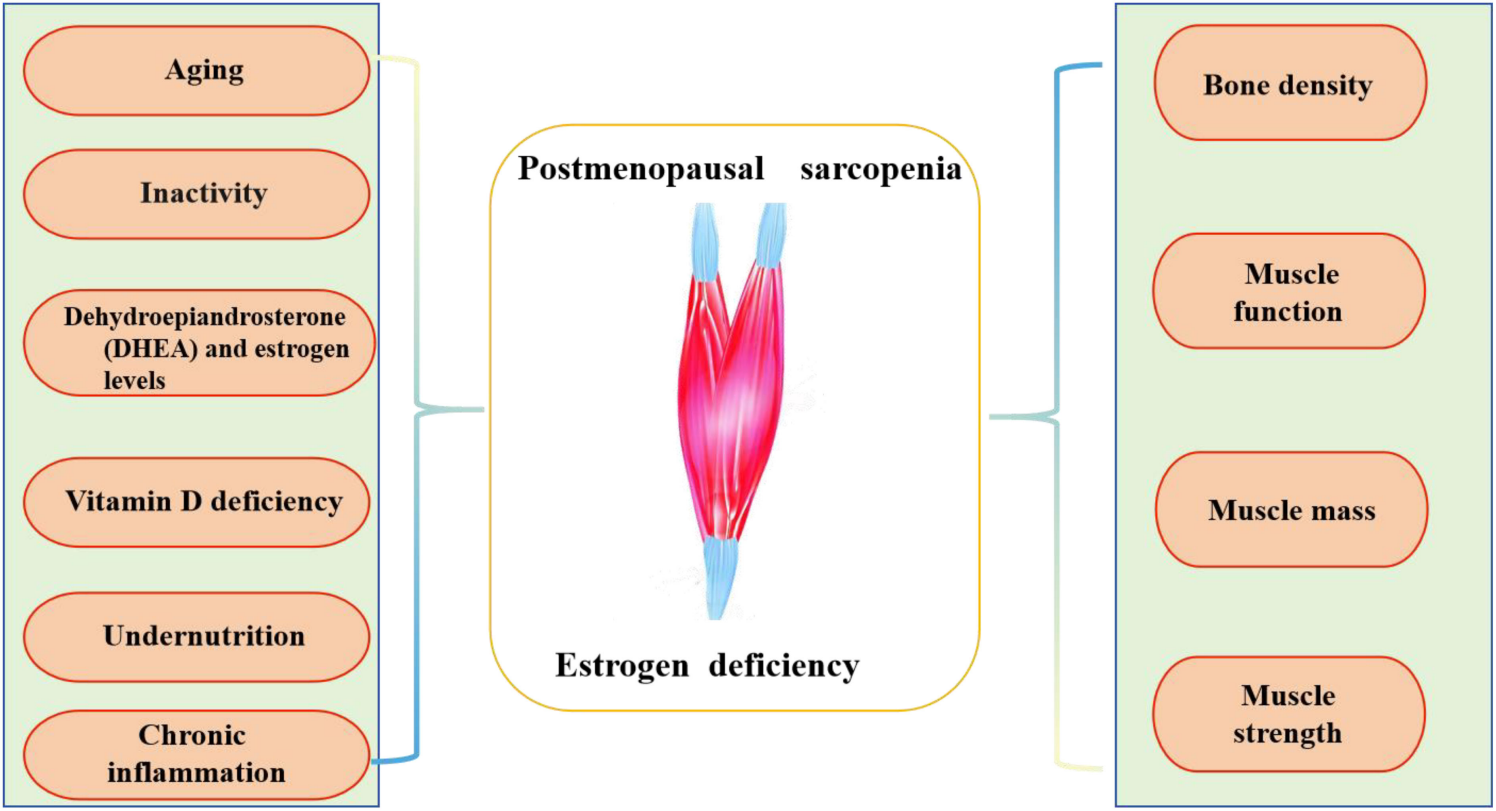
The description of postmenopausal sarcopenia.

The specific causes of PS involve various factors, such as age, decreased physical activity, muscle junction neurons, and levels of testosterone or estrogen, and dehydroepiandrosterone (DHEA) (37, 38). Chronic inflammation, insufficient vitamin D, and low nutrient, protein, or calorie intake (38–41) also contribute to this condition (as shown in **Figure 1**).

## 3 Effect of estrogen in skeletal muscle

### 3.1 The effect of estrogen on skeletal muscle physiological conditions

The influence of estrogen on muscle strength is multifaceted. Estrogen plays a crucial role in maintaining muscle mass and strength in physiological conditions (42, 43). Estrogen receptors, particularly ERβ, are found in skeletal muscles and mediate the effects of estrogen on muscle metabolism and function (19, 44). It has been hypothesized that estrogen may work through its receptors to improve muscle quality, preserving the structure and function of myosin, which is essential for muscle contraction and force generation (17).

Additionally, estrogen influences carbohydrate and fat utilization during rest and exercise, which can impact muscle energy supply and utilization (45). The process promotes muscle growth and repair by boosting the synthesis of muscle proteins and curbing their breakdown, which are crucial for these functions (14). Studies indicate that it can enhance muscle performance and minimize the likelihood of injuries in different musculoskeletal tissues such as muscles, tendons, and ligaments. While estrogen may enhance muscle function, it can also negatively affect the stiffness of tendons and ligaments, potentially increasing women’s susceptibility to injuries (46).

Experiments have demonstrated that in muscle-specific ER α knockout (KO) mice, the fatigue time of single muscle fibers is shorter than that of control mice. Moreover, *ESR1* knockdown in muscle cells impairs fatty acid oxidation (47), indicating that ER α plays a crucial role in skeletal muscle function.

Estrogen’s effect on muscle strength is also tied to its role in collagen synthesis and connective tissue health, which can influence the structural integrity and force transmission capacity of muscles (48).

Estrogen plays a role in muscle regeneration by influencing satellite cell function, which is crucial for muscle repair and growth after injury or exercise (49). It can also have antioxidant effects, helping to reduce oxidative stress in muscle tissues and supporting recovery (49, 50).

### 3.2 The effect of estrogen on muscle pathological conditions

With aging, the rate of muscle strength decline often exceeds the rate of decrease in muscle mass, indicating a decline in function or quality, muscle atrophy may also occur (8). Multiple human studies have demonstrated the efficacy of estrogen in mitigating the impact of menopause on skeletal muscle mass and strength (25, 51). Consequently, new treatment strategies targeting estrogen in non-reproductive organ tissues may offer therapeutic benefits by reducing the metabolic dysfunction associated with ovarian failure and estrogen deficiency. Reports have indicated changes in muscle tissue characteristics during menopause. Experimental results depict that the amount of non-contractile muscle tissue, such as intramuscular fat, is twice as high in postmenopausal women than in young women (7, 52).

In pathological conditions such as PS, the deficiency of estrogen be relevant to a significant loss of muscle mass and strength (15). Firstly, estrogen contributes to muscle mass maintenance, particularly in postmenopausal women where it can help counteract age-related muscle loss (45). This loss is attributed to the direct impact of estrogen on muscle fibers, satellite cell function, and muscle regeneration processes (45). Secondarily, estrogen deficiency can lead to a decrease in muscle mitochondrial function, which is essential for maintaining muscle health and energy metabolism (47). And a study involving ovariectomy and climbing training in adult female rats, estrogen treatment resulted in significantly larger muscle fiber sizes, including myosin heavy chain type II fibers, compared with sham surgery and group climbing training alone (53). This suggests that estrogen can enhance muscle hypertrophy induced by resistance training in postmenopausal women.

Experiments have demonstrated that skeletal muscle-specific ER α deficient mice exhibit reduced intensity and contractility (54). In ER α KO mice, the extensor digitorum longus displays eccentric and submaximal/maximal isometric impairment, while the soleus muscle demonstrates increased fatigue and weakened muscle strength recovery (55). These findings suggest that ER α mediates the beneficial effects of estradiol on muscle strength. In muscle-specific ERβ gene knockout (mERβKO) mice, there was no difference in treadmill performance compared with that in control mice. However, female mERbKO mice exhibited significant impairment in the absolute average maximum strength during grip strength testing. In addition, young female mERbKO mice exhibit a rapid and significant reduction in muscle mass, indicating that muscle-specific ERβ deficiency leads to decreased muscle mass and strength in female mice (56). Furthermore, estrogen’s neuroprotective effects are diminished in postmenopausal women, which can indirectly affect muscle function due to the close relationship between muscle and neuronal health (57).

### 3.3 The effect of estrogen on mitochondria metabolism in skeletal muscle

Skeletal muscles are the largest organs for energy metabolism and are primarily involved in oxidative metabolism and insulin-stimulated glucose uptake (58). Estrogen is linked to skeletal muscle metabolism; notably, *ESR1* levels in women with metabolic syndrome are decreased and negatively correlated with adipose tissue mass and fasting insulin levels (47). In terms of mitochondrial metabolism, estrogen is known to regulate the expression of genes related to antioxidant defense and energy balance within skeletal muscle. For instance, estrogen has been found to modulate the expression of estrogen receptors and antioxidant genes in mouse skeletal muscle, suggesting its role in maintaining muscle health and function (20, 59). Estrogen has a direct impact on mitochondrial function within skeletal muscle. It can improve mitochondrial bioenergetics by reducing membrane microviscosity and enhancing the efficiency of energy production (16, 20). This can lead to better muscle function and reduced fatigue, especially during high-intensity or endurance activities (20).

A recent research showed that female rats treated with estrogen demonstrated increased mitochondrial mass, antioxidant protection, and oxidative phosphorylation in the skeletal muscle, compared with male rats. Despite these benefits, the skeletal muscles of ovariectomized (OVX) female animals exhibit reduced oxygen consumption along with decreased expression of mitochondrial biogenesis-related genes and mitochondrial remodeling factors, which are associated with increased hydrogen peroxide production (49, 60).

Research findings indicate that postmenopausal women with estrogen deficiency have reduced mitochondrial content in their skeletal muscles, while estrogen replacement therapy can increase mitochondrial content (16). The scholar emphasizes that estrogen, through its receptors ERαand ERβ, stimulates the transcription of nuclear respiratory factor-1 (NRF-1), which in turn promotes the transcription of genes encoding mitochondrial proteins, including those of the electron transport chain (ETC). This leads to an increase in mitochondrial content and function (59). Besides, estrogen has been shown to increase the mitochondrial respiration in skeletal muscle cells (59).

### 3.4 The synergistic effect of testosterone and estrogen on muscle strength

Testosterone is a primary androgen with anabolic-androgenic properties. It is derived from cholesterol and is predominantly synthesized in men’s Leydig cells. In women, it is produced by the ovaries and adrenal zona fasciculata, each accounting for 25% of the total, through the conversion of progesterone. The remaining approximately 50% of testosterone is generated from circulating androstenedione (61). And, the study points out that the ovaries secrete androgens after menopause, however testosterone levels are not directly affected by the menopausal transition or the onset of menopause (61).

The study by Alexander et al. in 2022 indicates that testosterone can be converted to estrogen through the process of aromatization (43). This conversion is crucial as it may influence various health and performance aspects in postmenopausal women. In postmenopausal women, the decline in estrogen levels is associated with a decrease in muscle mass and strength (62). Then, the studies by Cauley (63), Kenny (64), and Dionne (62), have consistently observed a decline in muscle strength among postmenopausal women. This decline is a critical health concern that our manuscript addresses.

Estrogen, derived from testosterone, plays a role in muscle metabolism and function, which is crucial in preventing sarcopenia, the loss of muscle mass associated with aging (65). Furthermore, it has been shown that free testosterone levels, rather than total testosterone, correlate with lean body mass but not muscular strength in both pre- and post-menopausal women (43).

Ovariectomy (OVX) refers to the surgical removal of one or both ovaries and is used to assess the loss of ovarian hormones, primarily estrogen. It is the primary animal model for evaluating the effects of estrogen loss in females (66). Following ovariectomy, levels of other sex hormones, including testosterone and follicle-stimulating hormone also undergo corresponding changes (66), which it is necessary to take into consideration. The synergistic effect of testosterone on muscle mass and strength is a well-studied phenomenon, particularly as individuals age. According to a study published in 2011, testosterone level is positively related to muscle strength and physical performance in older men, not with muscle mass (67). The results found that testosterone levels were significantly associated with muscle strength and physical performance (68). This suggests that testosterone may have a direct effect on muscle fibers, potentially enhancing their function and the overall efficiency of muscle contractions.

Significant correlations were observed between skeletal muscle mass and factors such as BMI, age of initiation of estrogen replacement therapy, hand grip strength, lower limb strength and power, and testosterone levels; however, no correlation was found with estradiol levels (64). A systematic review and meta-analysis of 12 studies comprising 4474 postmenopausal women found that those who received estrogen-based hormone therapy lost less lean body mass compared with women who received no hormone therapy and women who received placebo, although this finding was not statistically significant (69).

To sum up, both testosterone and estrogen would affect muscle strength. The conversion of testosterone to estrogen in postmenopausal women is integral to maintaining muscle mass, regulating muscle protein synthesis, reducing muscle damage, and enhancing recovery and muscle repair. These factors collectively contribute to preserving muscle health and function in the face of age-related changes. These aspects highlight the importance of considering the role of testosterone and its conversion to estrogen in the context of postmenopausal health and well-being.

## 4 Molecular mechanisms of estrogen deficiency in sarcopenia

### 4.1 The molecular aspect of sarcopenia in postmenopausal women

Estrogen binds to its homologous receptors, estrogen receptors (ERs), ER α, and ER beta (ER β) in the nucleus or cytoplasm. Upon activation, these receptors translocate to the nucleus. Subsequently, ERs bind to estrogen-responsive elements (ERE) in the DNA sequence. Last, the coactivators or transcription factors were recruited to regulate the target genes (70). The receptor activates various signaling pathways, including insulin-like growth factor 1 (IGF-1), phosphorylated inositol-3 kinase/protein kinase B (AKT) pathways, growth differentiation factor 15 (GDF15), and the estrogen signaling influences the balance between mitochondrial fusion and fission. These pathways positively regulate the muscle satellite cells and promote protein synthesis (15, 59, 71).

Insulin-like growth factor 1 (IGF-1), is well-known for its anabolic effects on skeletal muscle (72). From Wan-Jung A Tsai findings indicate that diminished muscle IGF-1 protein levels could be implicated in the influence of estrogen on growth in immature, ovariectomized rats. Additionally, heightened muscle myostatin protein levels might contribute to the mediation of estrogen’s impact on growth (73). Estrogen can influence the expression and activity of IGF-1, and vice versa, creating a dynamic system that regulates muscle metabolism and adaptation to various physiological stimuli.

Estrogen deficiency in rodent models and postmenopausal women is associated with reduced AKT activation and increased expression of atrophy markers FOXO3 and MuRF1. Estrogen therapy reduces muscle atrophy, likely by activating the AKT-foxO1-MyoD pathway (74, 75).

Growth differentiation factor 15 (GDF15), a novel biomarker associated with sarcopenia, and its potential as a therapeutic target for muscle wasting in postmenopausal women (71). Estrogen and Growth Differentiation Factor 15 (GDF15) both play significant roles in the regulation of skeletal muscle metabolism and function. GDF15, a stress response cytokine and a member of the TGFβ superfamily, is increasingly recognized for its impact on muscle physiology and metabolism. It has been shown to enhance energy expenditure in muscle, which can contribute to weight loss and improved glycaemic control (76). Estrogen may modulate the expression of GDF15, which in turn affects muscle metabolism through its receptor GFRAL. This signaling can influence appetite, energy balance, and muscle bioenergetics, highlighting the potential for estrogen to indirectly affect muscle function via GDF15 regulation (76).

Proper mitochondrial dynamics are crucial for preserving the health of muscle cells and averting muscle-related ailments (59). Estrogen contributes significantly to antioxidant defense and metabolic control by diminishing the generation of mitochondrial reactive oxygen species (ROS) in skeletal muscles, modulating the genes that govern glucose and lipid metabolism, and sustaining the functionality of mitochondria and the structural integrity of muscle fibers. This is particularly prevention muscle atrophy, especially under conditions such as aging or menopause (59). It is worth mentioning that estrogen signaling in skeletal muscle mitochondria intersects with other important pathways, such as the AMP-activated protein kinase (AMPK) signaling, to regulate muscle metabolism and energy balance (11).

Above all, estrogen’s effects on muscle metabolism are complex and wide-ranging, impacting muscle mass, strength, mitochondrial function, and regenerate after injury. These effects emphasize the importance of estrogen in maintaining musculoskeletal health and performance.

### 4.2 The regulation of estrogen on muscle bioenergetic signaling

In skeletal muscle, estrogen signaling has been shown to influence muscle mass and function. For instance, muscle-specific estrogen receptor α-deficient mice (MERKO) exhibit muscle mitochondria dysfunction. It is characterized by reduced oxygen consumption rates and increased production of ROS, indicating a role for estrogen in maintaining mitochondrial health (59).

Furthermore, estrogen can stimulate nuclear respiratory factor-1 transcription, which interacts with coactivators like peroxisome proliferator-activated receptor gamma coactivator 1 alpha (PGC1-α) to regulate nuclear-encoded mitochondrial genes (59, 77). This pathway can influence mitochondrial bioenergetics, oxygen consumption rate, and extracellular acidification (59, 78).

In summary, estrogen regulates muscle bioenergetic signaling through a complex interplay of nuclear and mitochondrial-mediated events, including the regulation of gene transcription by ERs, the modulation of metabolic gene expression, and the influence on mitochondrial function and bioenergetics. These actions collectively contribute to maintaining muscle mass and function.

## 5 Hormone Replacement Therapy (HRT) and sarcopenia

### 5.1 Effects of HRT on muscle mass and function

Hormone Replacement Therapy (HRT) has been a topic of great concern, particularly in the context of sarcopenia (37). Some research suggests that HRT has no significant impact on these parameters, other studies propose that estrogen and synthetic HRT could exert anabolic effects on skeletal muscle (69). While a discrepancy exists in the literature regarding the effects of HRT on muscle strength and mass, as well as its role in combating sarcopenia (79).

HRT has been shown to have a positive impact on muscle mass and function in postmenopausal women. Estrogen, the primary hormone replaced in HRT, plays a crucial role in maintaining muscle mass and strength (18, 63). A study by Kim SW and Kim R. demonstrated an association between hormone therapy and a reduced prevalence of sarcopenia in postmenopausal women, highlighting the potential benefits of HRT in combating age-related muscle deterioration (17).

There is clear literature support for the use of estrogen replacement therapy to enhance muscle strength (43, 63, 80, 81). Although a study from suggests that tibolone appears to offer more benefits than HRT in treating body composition and muscle strength in postmenopausal women (81).

### 5.2 Types of HRT and their impact on sarcopenia

Now the three main types of HRT are estrogen-single therapy and estrogen with added progesterone with either continuous or cyclic use of progestin (82, 83). From studies have clarified that estrogen-only therapy is recommended for women who have had a hysterectomy. The usual oral estrogen types used include estradiol and estrogen. The starting dose is usually 0.5-2 mg/d of estradiol (83).

In medical practice, estrogen should be used with progesterone or bazedoxifene, a selective estrogen receptor regulator (SERM), once a day to minimize the risk of malignancies in women with an intact uterus (83, 84). It can reduce vasomotor symptoms in postmenopausal women and harm women’s bone mass, endometrium and breast safety. The dose usually is estrogen 0.45 mg plus bazedoxifene 20 mg/d (85).

Third, bazedoxifene, which combines equine estrogen, is also being considered as an alternative treatment option for postmenopausal women in the uterus. This would avoid excluding a pill containing progesterone when it is contraindicated to take progesterone. Specifically, conjugated equine estrogens (0.625 mg/d) and medroxyprogesterone acetate (2.5 mg/d).

It should be added that in postmenopausal women over the age of 65, low-dose hormone therapy appears to confer greater risk reduction compared to medium and high doses. Additionally, vaginal or transdermal administration is more effective than oral formulations, and the use of E2 (estradiol) is more effective than conjugated equine estrogen (86).

These forms of HRT have been associated with improved muscle mass and strength in postmenopausal women, although the specific impact on sarcopenia may vary depending on individual menopausal status and physical activity levels (82). Mass pre-thrombotic status screening of women prior to the first use of prescription hormone replacement therapy is therefore particularly recommended. To minimize cardiovascular problems associated with HRT.

### 5.3 Safety and side effects of HRT in postmenopausal women

HRT can provide relief from menopausal symptoms and improve muscle mass and function, but there are also risks. Potential side effects include an increased chance of developing serious conditions like breast cancer and heart disease, particularly in women who start HRT at an older age or more than 10 years after menopause (87). A randomized controlled trial from Jacques E Rossouw teams, showed the overall risks of combining estrogen and progesterone outweighed the health benefits followed for an average of 5.2 years (88).

Safety concerns surround ERT, with some studies suggesting an elevated risk of serious health is like heart disease, stroke, and thrombosis, especially among women who begin treatment at an advanced age or well after menopause. It is essential for healthcare providers to weigh the benefits and risks of HRT on an individual basis, considering factors such as the patient’s symptoms, medical history, and overall health (86, 89).

In particular, note that HRT can exert an important role in mitigating sarcopenia in postmenopausal women by preserving muscle mass and function (85). However, the decision to initiate HRT should be made seriously and should be tailored to the individual’s needs and health status.

## 6 Conclusion

**Figure 2:**
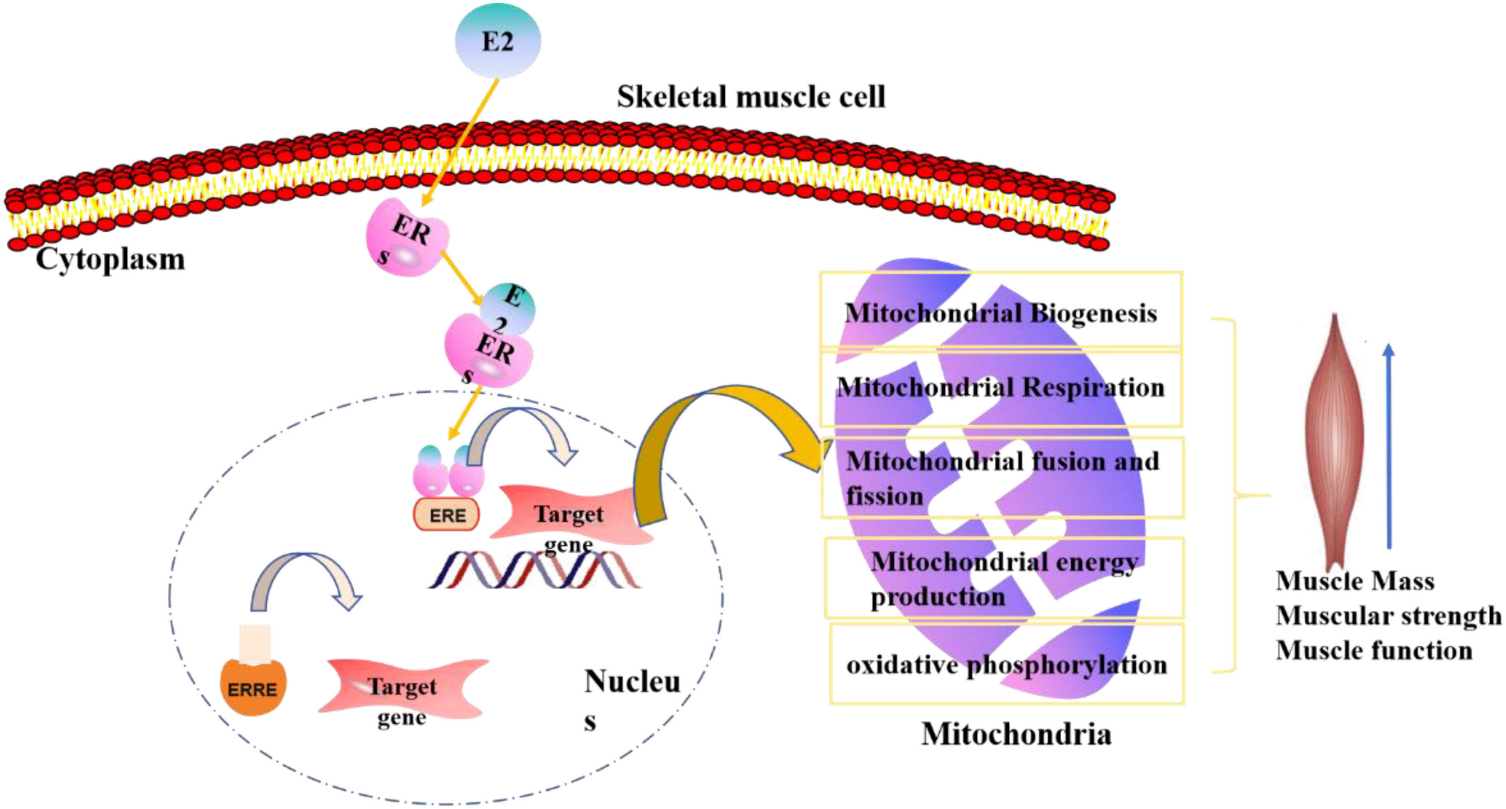
The estrogen regulation model of estrogen functions in postmenopausal sarcopenia, mainly concentrate on skeletal muscle and mitochondrial regulation.

In this review, we have examined the intricate relationship between PS and estrogen, highlighting the hormone’s multifaceted impact on skeletal muscle. Estrogen plays a crucial role in preserving muscle mass, particularly through its influence on mitochondrial metabolism and synergistic effects with testosterone on muscle strength. The molecular mechanisms by which estrogen deficiency contributes to sarcopenia have been elucidated, emphasizing the regulation of muscle bioenergetic signaling (as shown in **Figure 2**).

Hormone Replacement Therapy (HRT) emerges as a potential intervention for mitigating sarcopenia, with varying effects on muscle mass and function depending on the type of HRT. There is no universal approach to ERT dosage.

This article suggests opting for the minimal effective dose to mitigate risks, yet the decision should be personalized, considering the patient’s health profile and therapeutic objectives.

To sum up, the ERT in postmenopausal care is unclear and warrants additional research. Its application should be carefully considered against potential risks and should always be overseen by a healthcare provider.
